# Quorum sensing *N*-acyl homoserine lactones-SdiA enhances the biofilm formation of *E. coli* by regulating sRNA CsrB expression

**DOI:** 10.1016/j.heliyon.2023.e21658

**Published:** 2023-10-29

**Authors:** Shebin Zhang, Yurong Shu, Weizheng Zhang, Zhenjie Xu, Youqiang Li, Song Li, Qiwei Li, Rui Xiong, Yifei Long, Jianping Liu, Yunyan Zhang, Cha Chen, Yang Lu

**Affiliations:** a Department of Laboratory Medicine, The Sixth Affiliated Hospital of Guangzhou Medical University, Qingyuan People's Hospital Guangzhou, Qingyuan, PR China; b Department of Laboratory Medicine, The Second Affiliated Hospital of Guangzhou University of Chinese Medicine, Guangzhou, PR China; c The Second Clinical College, Guangzhou University of Chinese Medicine, Guangzhou, PR China; d Department of Laboratory Medicine, Guangzhou No.11 People's Hospital, Guangzhou Cadre Health Management Center, Guangzhou, PR China; e Department of Laboratory Medicine, The Affiliated Hexian Memorial Hospital of Southern Medical University, Guangzhou, PR China; f Affiliated Stomatology Hospital of Guangzhou Medical University, Guangzhou, PR China

**Keywords:** Biofilm formation, CsrB, *Escherichia coli*, N-acylhomoserinelactones, SdiA

## Abstract

As an important virulence phenotype of *Escherichia coli*, the regulation mechanism of biofilm by non-coding RNA and quorum sensing system has not been clarified. Here, by transcriptome sequencing and RT-PCR analysis, we found CsrB, a non-coding RNA of the carbon storage regulation system, was positively regulated by the LuxR protein SdiA. Furthermore, β-galactosidase reporter assays showed that SdiA enhanced promoter transcriptional activity of *csrB*. The consistent dynamic expression levels of SdiA and CsrB during *Escherichia coli* growth were also detected. Moreover, curli assays and biofilm assays showed *sdiA* deficiency in *Escherichia coli SM10λπ* or *BW25113* led to a decreased formation of biofilm, and was significantly restored by over-expression of CsrB. Interestingly, the regulations of SdiA on CsrB in biofilm formation were enhanced by quorum sensing signal molecules AHLs. In conclusion, SdiA plays a crucial role in *Escherichia coli* biofilm formation by regulating the expression of non-coding RNA CsrB. Our study provides new insights into SdiA-non-coding RNA regulatory network involved in *Escherichia coli* biofilm formation.

## 1 Introduction

*Escherichia coli* (*E. coli*) is an opportunistic bacterial pathogen, which usually causes urinary tract infection and surgical implantation infection [1]. The main cause of chronic bacterial infection is biofilm, which plays an important role in colonization, drug resistance and immune resistance of bacterial communities [2]. Biofilm formation is regulated by a variety of complexes signaling pathways due to the change of living environment, among which the carbon storage regulation (Csr) system is an important one. The Csr system also known as the Rsm (repressor of secondary metabolites) system, is an non-coding RNA (sRNA) system that has been previously found to be related to the bacterial carbon sources metabolism, consisting of transcription regulator protein CsrA, and sRNA CsrB and CsrC [3]. CsrA is a RNA-binding protein that regulates gene expression through destabilizing or stabilizing target transcription, primarily recognized as a function in inhibiting glycogen metabolism, peptide transport, and biofilm formation, respectively [[4], [5], [6]]. The sRNA CsrB and CsrC have recognition sequences of CsrA, which can combine with CsrA into a globular ribonucleoprotein complex and sequester CsrA away from other transcripts [7]. Some studies showed that the formation of bacterial biofilm was defective after the deletion of CsrB, which plays an important role in the Csr system and promotes biofilm formation by interacting with CsrA [[8], [9], [10]].

Quorum sensing (QS) is a process in which bacteria monitor their population density by producing and sensing extracellular signals, promote mutual communication between bacteria to adapt to environmental changes and coordinate group behaviors [11]. AHLs production is absent in *E. coli*, which only has the luxR-type protein SdiA [12]. Case et al. described the phenomenon of non-AHL producing microorganisms binding and utilizing AHLs produced by other organisms as eavesdropping [13]. As a transcription factor, SdiA can bind to DNA to regulate gene transcription in the absence of AHLs, while the presence of AHLs enhances DNA-binding affinity and stability of SdiA, thus regulate gene expression in a SdiA-dependent manner [14]*.* For example, using C4-HSL and 3-oxo-C12-HSL, we have previously discovered that they can enhance the regulatory function of SdiA [15]. As an adaptive monitor, QS system can regulate biofilm and other virulence factors to alter survival strategies [16,17]. In spite of this, there is a great deal of uncertainty about the role of SdiA in biofilm formation mechanisms. Interestingly, in recent years, some sRNAs related to the QS system have been gradually discovered and found to be involved in the regulation of biofilm generation [18]. Thus, how bacteria regulate the formation of biofilm through QS system, especially QS-related sRNA, needs further research.

Here, the role of SdiA in the Csr system and its regulatory mechanism on the formation of biofilms in *E. coli* was explored. Firstly, the effect of *sdiA*-knockout and -overexpression on CsrB was confirmed through β-galactosidase reporter analysis, RT-PCR and transcriptome. Subsequently, curli and biofilm assays showed that CsrB was involved in SdiA-regulated biofilm formation, the phenomenon was enhanced by exogenous AHLs. Last, we also detected the dynamic changes of genes expression during biofilm formation to prove the correlation between them. Our findings provide further evidence and views for exploring the influence of SdiA on biofilms.

## 2 Materials and methods

### 2.1 Bacterial strains, plasmids and growth conditions

The strains and plasmids used in this study are listed in Supplementary Table S1. *SM10λπ* and *BW25113* strains are often used to study the regulatory mechanism of *E. coli* [19,20], and the DH5α strain was used to construct overexpression vector. The details for construction of strains with *sdiA*-deficiency or -overexpression have been showed in our previous study [21]. Luria-Bertani (LB) medium was used to culture the bacteria and the following concentrations of AHLs or antibiotics was added if necessary: chloramphenicol (*Cm*, 16 μg/ml), ampicillin (Amp, 100 μg/ml), C4-HSL or 3-oxo-C12-HSL (40 μM each, DMSO as a solvent for AHLs). This AHL concentration can effectively promote the function of SdiA [15,21].

### 2.2 RNA sequencing

The indicated strains was cultured to mid-exponential phase (about 0.5 McFarland standard). RNA was then extracted with RNAprep Pure Cell/Bacteria Kit (Tiangen, Beijing, China). BGI Company conducted the entire sequencing. The CPM (Counts per million) method was utilized to compute the expression of Unigene.

### 2.3 Real-time PCR

In the experiment of detecting gene expression by long-term incubation, 30 μl *SM10λπ* wild-type strain cultures were added into a 3 ml LB for 37 °C incubation, the planktonic group was incubated in 15 ml tube at 200 rpm, and the adhesive group was incubated in a 6-well plate. Total RNA was extracted and then reverse transcripted into cDNA, which was used for qPCR to detect gene expression levels. To evaluate the expression levels of target genes, 2^−ΔΔCt^ method was used and *rpoD* was used as the internal control gene. Supplementary Table S2 has listed the sequence of primers for this experiment.

### 2.4 β-Galactosidase assays

The reporter plasmid pQF50 (with gene *lacZ* but without promoter) was used for *csrB* promoter analysis. The DNA fragment predicted as a *csrB* promoter was cloned and inserted into *Hin*dIII/*Bam*HI sites upstream of *lacZ* in pQF50. According to the Miller's method, the β-Galactosidase activity assays were performed on *E. coli BW25113* and AHLs were added in medium while the control group was treated with equivalent DMSO.

### 2.5 Biofilm assays and curli assays

Cells of *E. coli SM10λπ* and *BW25113* were grown overnight at 37 °C 200 rpm, then subcultured to exponential phase and diluted to 1 × 10^7^ cfu/ml. For biofilm assays, 30 μl cultures were added to a 6-well plate containing 3 ml LB with AHLs/DMSO and then incubated at 37 °C for 24 h. Crystal violet staining was used to quantify the microorganism biofilm according to the previous study [23]. For curli assays, 5 μl cultures were inoculated on LB-curli agar (10 g/l tryptone, 5 g/l yeast extract, 15 g/l agar, 20 μg/ml Congo red, 10 μg/ml Coomassie Brilliant Blue, 1 mM IPTG) and then grown at 28 °C for 48 h [22,23].

### 2.6 Statistical analysis

The mean ± standard deviation (SD) was used to express the data from multiple independent experiments conducted at least in triplicate. Data analysis will use Student's *t*-test between groups or one-way ANOVA for differences statistical analysis. * means the differences with a value of 0.01 < *P* < 0.05, ** means the differences with a value of *P* < 0.01, *** means the differences with a value of *P* < 0.001.

## 3 Results

### 3.1 Identification of AHL-SdiA-regulated sRNA in *E. coli*

To identify sRNAs regulated by SdiA in *E. coli*, we performed transcriptome sequencing of the wild-type strain of *SM10λπ* (WT), *sdiA* gene-deficiency strain (*ΔsdiA*), and *sdiA*-overexpression strain with *sdiA* mutation (*ΔsdiA/*SdiA). We also checked the influence of AHLs on the expression of sRNA in the above strains. Distinguishingly, the results showed that among most of the sRNAs annotated in NCBI database, CsrB was strongly expressed in WT strain and down-regulated in *sdiA*-deficiency strain, while restored in *sdiA*-overexpression strain (Table 1).

**Table 1:** Transcript level of genes in SM10λpir *sdiA* deficient and complemented strains.

Gene	DMSO			AHLs		
	WT	Δ*sdiA*	Δ*sdiA*-SdiA	WT	Δ*sdiA*	Δ*sdiA*-SdiA
*csrB*	2201.224	1496.481	2283.274	3044.125	1425.168	2191.556
*csrC*	100.841	83.679	110.784	79.28	67.014	96.941
*uvrY*	135.006	136.765	109.245	152.491	124.185	116.469
*barA*	182.112	186.28	132.866	172.685	185.045	152.69
*rnpB*	18088.964	21077.586	21794.672	19485.191	16755.917	21803.649
*gcvB*	97.437	92.145	75.824	65.622	67.476	66.813
*ssrS*	42.406	39.972	47.079	42.016	21.26	37.309
*glmZ*	14.729	15.147	11.498	9.502	11.092	11.396
*ryfA*	44.348	46.002	40.554	45.727	42.519	47.3
*ryeA*	10.197	9.397	22.219	9.799	10.784	19.825
*esrE*	1.779	2.191	1.931	2.442	2.255	3.317
*sgrS*	4.856	2.244	6.837	3.86	2.927	6.713
*rttR*	0	0.14	0.466	0	0	0.312
*ryfD*	NA	NA	NA	NA	NA	NA
*arcZ*	NA	NA	NA	NA	NA	NA
*psrO*	NA	NA	NA	NA	NA	NA
*sibE*	NA	NA	NA	NA	NA	NA
*tff*	NA	NA	NA	NA	NA	NA
*micA*	NA	NA	NA	NA	NA	NA
*omrA*	NA	NA	NA	NA	NA	NA
*sibB*	NA	NA	NA	NA	NA	NA
*eyeA*	NA	NA	NA	NA	NA	NA
*spf*	NA	NA	NA	NA	NA	NA
*rybB*	NA	NA	NA	NA	NA	NA
*mgrR*	NA	NA	NA	NA	NA	NA
*isrC*	NA	NA	NA	NA	NA	NA
*sibD*	NA	NA	NA	NA	NA	NA
*sibC*	NA	NA	NA	NA	NA	NA
*fnrS*	NA	NA	NA	NA	NA	NA
*rydB*	NA	NA	NA	NA	NA	NA
*gadY*	NA	NA	NA	NA	NA	NA

The calculation of Unigene expression uses CPM method (Counts per million).

Quantitative RT-PCR was then used to analyze the expression of CsrB in *SM10λπ* based on the evident change in transcriptome data, as well as another *E. coli* strain *BW25113*. Conformably, deficiency of *sdiA* in both *SM10λπ* and *BW25113* resulted in downregulation of CsrB, which was reversed by restoration of *sdiA*. Treatment with AHLs enhanced this regulatory function of SdiA (Fig. 1). In summary, these results suggest that expression of *csrB* is positively regulated by SdiA, and the effect was enhanced by AHLs.

**Fig. 1:**
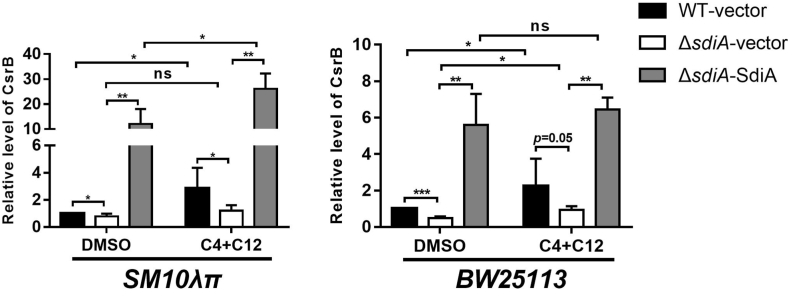
SdiA promotes sRNA CsrB expression in both *E. coli SM10λπ* and *BW25113* strains, and this promotion effect is enhanced by AHLs. *E. coli SM10λπ* or *BW25113* (WT-vector) and the *sdiA*-deficient strains (*ΔsdiA*-vector) carrying pSTV28, the *sdiA*-overexpression strains (*ΔsdiA*-SdiA) carrying pSTV28-*sdiA*, were subcultured in LB for 2 h, followed by RT-PCR analysis; the *rpoD* gene was used as an internal control. The concentration of C4-HSL and 3-oxo-C12-HSL was 40 μM each, while the DMSO group was added with the same amount of solvent DMSO as control. *, *P* < 0.05; **, *P* < 0.01; ***, *P* < 0.001.

### 3.2 SdiA regulates transcription of *csrB*

Subsequently, the impact of SdiA on the transcription activity of the *csrB* promoter region was assessed. The DNA fragment containing promoter was cloned and inserted into the corresponding region of the pQF50 reporter plasmid to construct a *lacZ* reporting system. Then the plasmid was transformed into *BW25113* strain which cannot produce endogenous β-galactosidase. Compared to the blank group, *csrB*-*lacZ* showed a high β-galactosidase activity (Fig. 2A). What counts is that mutation of *sdiA* impaired the activity of *csrB*-*lacZ*, which was rescued by overexpression of SdiA. Moreover, treatment with AHLs also enhanced the promoter activity of *csrB* (Fig. 2B). These findings were in line with the quantitative RT-PCR outcome mentioned earlier, and demonstrate that SdiA can promote *csrB* transcription by activating its promoter.

**Fig. 2:**
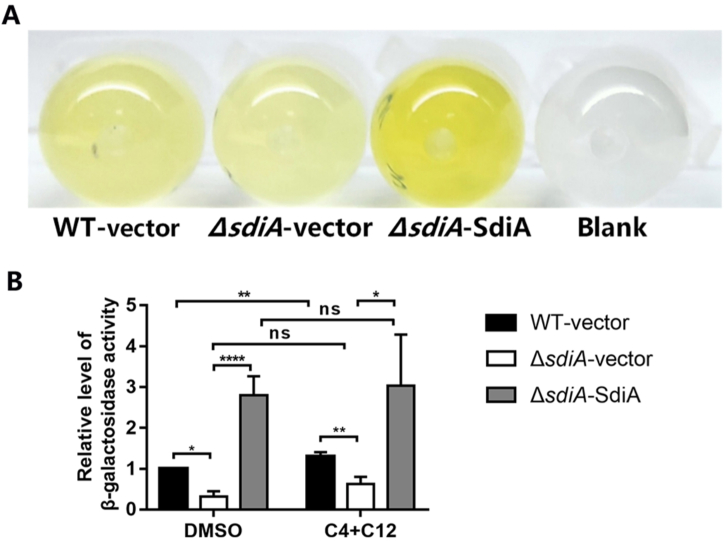
The promoter activity of *csrB* is positively regulated by SdiA and enhenced by AHLs. The *BW25113* strains carrying the reporter pQF50 (Blank) or pQF50-P*csrB* combined with pSTV28 or pSTV28-*sdiA* were grown to mid-log phase, subjected to β-galactosidase activity assay according to the modifified Miller's method. The concentration of C4-HSL and 3-oxo-C12-HSL was 40 μM each, while the DMSO group was added with equivalent solvent DMSO as control. Values are mean ± SD of at least three independent experiments. *, *P* < 0.05; **, *P* < 0.01; ****, *P* < 0.0001.

### 3.3 SdiA enhances biofilm formation of *E. coli* through CsrB

We further evaluated whether CsrB was involved in SdiA-regulated biofilm formation. Curli and biofilm assays were performed in the above *SM10λπ* strains with different *sdiA* expression. Curli are extracellular proteinaceous fibers that are important for biofilms formation [24]. The result showed that curli stain in both wild-type and *sdiA* mutant strain was almost no difference, nevertheless, overexpression of SdiA obviously promoted curli formation (Fig. 3). Similar result was also observed for biofilm formation (Fig. 4A). Moreover, under the treatment with AHLs, biofilm level of WT was elevated. On this basis, deficiency of *sdiA* in *E. coli* resulted in impaired biofilm formation, which was rescued by overexpression of SdiA (Fig. 4B). Then CsrB was overexpression in *sdiA*-deficiency strain (Δ*sdiA*-CsrB), which showed increased biofilm formation compared with *sdiA*-deficiency strain (Fig. 4C). These results indicated that SdiA enhances the biofilm formation though CsrB.

**Fig. 3:**
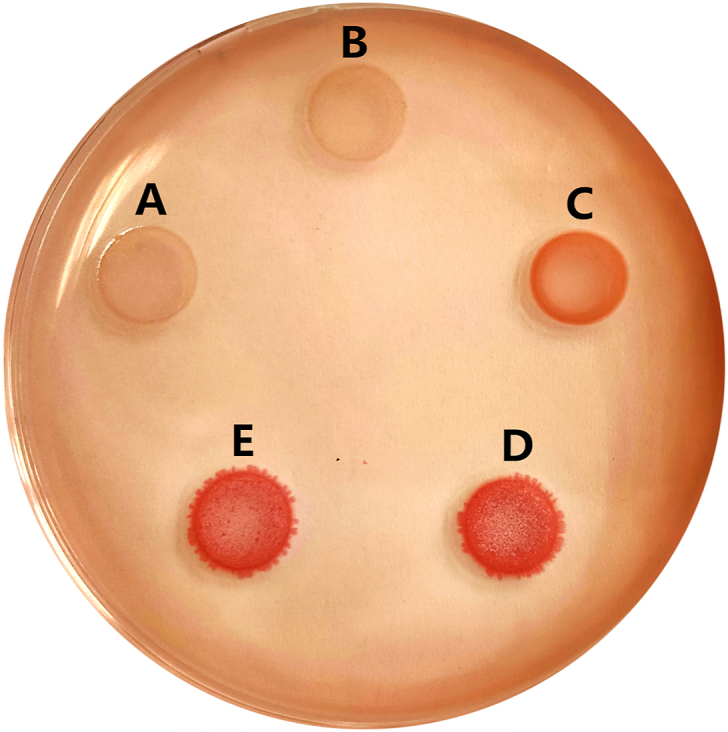
SdiA improves curli formation. 5 μl 1 × 10^7^ cfu/ml exponential phase cultures were inoculated on LB-curli agar and grown at 28 °C for 48 h. The strains used are: *E. coli SM10λπ* carrying pSTV28 (A), *sdiA*-deficient strains carrying pSTV28 (B), *sdiA*-deficient strains carrying pSTV28-*sdiA* (C), *E. coli SM10λπ* carrying pSTV28-*csrB* (D) and *sdiA*-deficient strains carrying pSTV28-*csrB* (E).

**Fig. 4:**
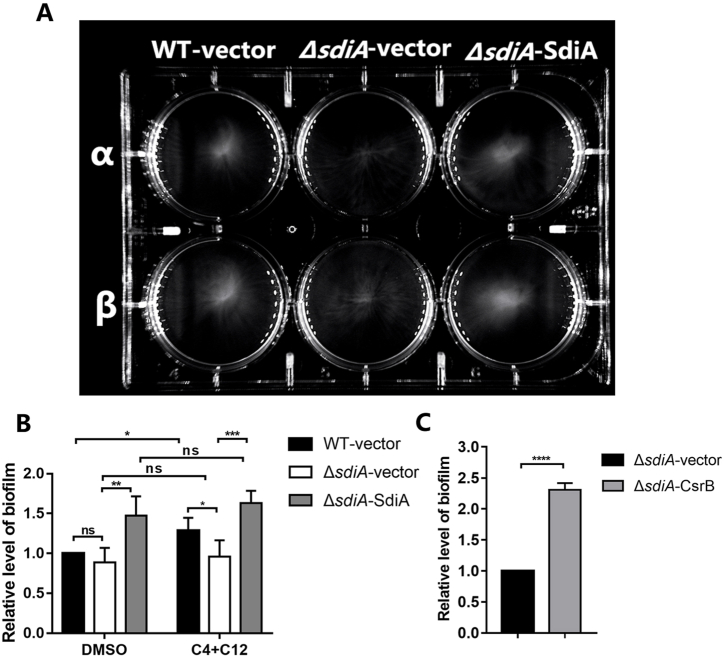
SdiA improves biofilm formation and enhenced by AHLs. (A) *E.coli SM10λπ* (WT-vector) and the *sdiA*-deficient strains (*ΔsdiA*-vector) carrying pSTV28, the *sdiA*-overexpression strains (*ΔsdiA*-SdiA) carrying pSTV28-*sdiA*, were subcultured to exponential phase and diluted to 1 × 10^7^ cfu/ml, then 30 μl cultures were added to a 6-well plate containing 3 ml LB with DMSO (α)/AHLs (β) and then incubated at 37 °C for 24 h. (B) Follow the instructions above, crystal violet staining was used for biofilm quantification. (C) pSTV28-*csrB* was transformed into *sdiA*-deficient strains and compared with *sdiA*-deficient strains in biofilm formation. Values are mean ± SD of at least three independent experiments. *, *P* < 0.05; **, *P* < 0.01; ***, *P* < 0.001; ****, *P* < 0.0001.

**Fig. 5:**
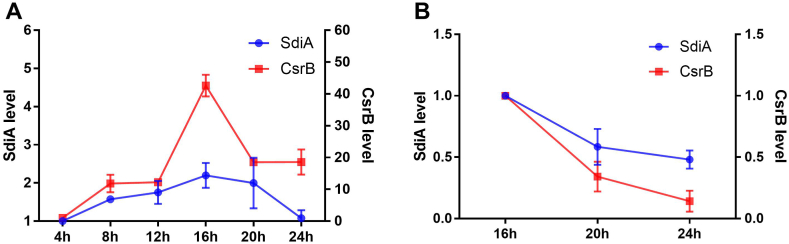
Synergistic changes of SdiA and CsrB during bacterial growth in different state. 30 μl *SM10λπ* wild-type strain cultures were added into a 3 ml LB for 37 °C incubation. (A) The cultures were incubated at 15 ml tube at 200 rpm to keep the bacteria in planktonic state. (B) The cultures were incubated at 6-well plate to keep the bacteria in adhesive state. The cultures will be collected and followed by RT-PCR analysis. Values are mean ± SD of at least three independent experiments.

Next, we explored the dynamic expression of SdiA and CsrB in planktonic and adhesive culture model. The planktonic state group was cultured in the shaking medium, both the expression of SdiA and CsrB increased first, reached the peak at 16 h and then decreased (Fig. 5A). For the adhesive state group, bacteria were cultured in a static 24-well flat-bottomed plates, in consideration of little biofilm formation in early stage, the experiment was conducted after 16 h’ culture, in which expression of CsrB decreased with SdiA, indicating that expression of CsrB and SdiA were also closely related during biofilm formation process (Fig. 5B). RT-PCR results suggest that the expression level of CsrB was synergistic with SdiA.

## 4 Discussion

As a LuxR homolog, SdiA detects the AHLs produced by other microorganism. Most recent publications regarding SdiA have documented its regulatory mechanism for protein-encoding genes as a transcriptional regulator [24]. Here, we present our findings on the identification of SdiA-regulated sRNA. Our results indicated that CsrB is positively regulated by SdiA, and the enhancement effect was improved after adding the signal molecule of SdiA, AHLs. Nevertheless, in transcriptome sequencing, the expression of CsrB did not recover to the level of wild-type strains after the addition of AHLs, but the overexpression strain was a complement to SdiA constructed on the basis of *sdiA*-deficient strains, and compared to *sdiA*-deficient strains, it also showed the trend of reversion, so we believe that SdiA plays an crucial role in the regulation of CsrB expression. Besides, CsrC, which functioned similarly to CsrB, also showed a medium expression change in *sdiA* gene-deficient and -overexpression strain, and there was still a regulatory relationship after the addition of AHLs, but the level did not increase like CsrB but slightly decreased, which is very worthy of further discussion. This suggests that SdiA may regulate the Csr system and thus affect the corresponding phenotypes. In some previous reports, SdiA can regulate the expression of CsrB through UvrY/BarA pathway. However, our transcriptome sequencing and qPCR results show that the change of UvrY/BarA is not obvious (Table 1 and Fig. S1), which indicates that there may be another pathway and the relationship between the addition of AHLs and the regulation of CsrB and biofilm expression is also very worthy of study. The reason may be that the strain we used is inconsistent with that previously reported. It should be pointed out that a few sRNAs were screened in our normal germiculture model, this may be explained by that bacteria changed the expression of sRNA through sensing the environment. Further studies are needed to figure out the relationship between SdiA and sRNA systems under different environmental stress.

Many SdiA regulon members have been described up to now [12,25]. It is considered that some genes with particular sequences 5′-AAAAG(N8)GA AAA-3’ (SdiA-box) in promoter could be the potential targets of SdiA [26]. In this study, β-galactosidase activity assay demonstrated that SdiA can activate the *csrB* promoter, however, SdiA-box was not found in the promoter sequence of *csrB*. There may be other recognition site of SdiA or other SdiA-regulated transcription factor that affected *csrB* expression, the specific mechanism needs to continue to be verified. When we tested the transcriptional regulation of SdiA on CsrB, the results obtained by the reporting system were more significant than those obtained by transcriptome sequencing. The possible reason is that the reporting system alone overexpressed the *csrB* promoter and SdiA, so it could more specifically detect the regulatory effect of *csrB* promoter, but it might also artificially amplify this effect, because the number of copies of the *csrB* promoter at the bacterial genome level is not so much as overexpression and may be affected by a variety of transcription factors. Therefore, the reporting system can only show that SdiA can indeed enhance the transcriptional activity of the *csrB* promoter, but transcriptome data can more truly reflect the impact of SdiA on the expression of CsrB. Previous studies have reported that in the presence of AHLs, DNA-binding affinity of SdiA was significantly increased [27]. This was consistent with our observation that SdiA regulated CsrB expression more strongly in the presence of AHL. In addition, as the solvent of AHLs, DMSO and methanol were also confirmed to have no effect on *csrB* expression. The expression of CsrB in different AHLs concentrations and solvent DMSO or methanol can be seen in Supplementary Fig. S2 and Fig. S3 by β-galactosidase activity assay and RT-PCR.

Bacterial biofilm-related microorganisms are widely considered to be more tolerant to the external environment, and most of soft tissue slow inflammation, infectious diseases and chronic diseases with latent tendency are related to bacterial biofilm formation [28]. Thus, it is essential to search the mechanism of biofilm formation. Our results here reveal that SdiA promotes the production of crucial adhesive organelles curli fimbriae (Fig. 3) and improves the biofilm formation level (Fig. 4A and B), furthermore, CsrB has also been identified as a key component for biofilm formation (Fig. 4C). Interestingly, the regulation of SdiA in biofilm formation can also be enhanced by QS signaling molecule AHLs, which *E. coli* cannot produce by itself, so this promotion suggests the mutual adaptation or competition between *E. coli* and other microorganisms in the environment. All these phenotypic results are consistent with the above SdiA-CsrB regulatory relationship. In order to confirm this regulatory relationship, we explored the changes in gene expression of *E. coli* during biofilm formation, and found out that both SdiA and CsrB maintained a cooperative expression trend during the entire culture process, reached the peak at 16 h and then decreased (Fig. 5). SdiA was originally found to control cell division by regulating *ftsQAZ* [29,30]. When the bacteria grow to the later stage, due to the increase of SdiA inhibitory transcription factor, the accumulation of extracellular factor *N*-(3-oxohexanoyl)-HSL and the change of culture conditions, the transcription of SdiA will be reduced [29,31], which may also be the reason why cell proliferation reaches a relatively slow plateau is consistent with our results, and with it, CsrB also begins to decrease. To sum up, our results will enable the discovery and supplementation of more information about whether SdiA functions through sRNA, thus enabling the bacteria to adapt to environmental stress and regulate viability. Since both SdiA and biofilm formation are members of a complex regulatory network, more studies are needed to comfirm the role of SdiA-AHL signaling pathway in biofilm formation in different conditions and discover more pathways and components.

## 5 Conclusion

Biofilm is one of the important virulence of microorganisms and is the cause of clinical drug resistance and difficult to treat infections. Quorum sensing and sRNA are currently factors that affect microbial activity, and their regulatory mechanisms for biofilm formation will be explored in this study. *E.coli* SdiA is a LuxR-like quorum sensing signaling molecular receptor. We constructed SdiA knockout and overexpression strains, then found that SdiA was positively correlated with sRNA CsrB expression levels related to bacterial metabolism and biofilm in transcriptome sequencing and quantitative RT-PCR. Besides, our β-galactosidase assays also revealed that the transcription of CsrB was affected by SdiA, and the above effects were enhanced by the signaling molecule AHLs. Biofilm assays and curli assays showed that SdiA can promote biofilm formation. Last, the result demonstrated that the transcription levels of CsrB and SdiA were synergistic during long-term culture in the planktonic or adhesive state, indicating that the expressions of the two are also closely related in the biofilm formation process. This study provides new insights into SdiA-sRNA regulatory network involved in *E.coli* biofilm formation, but many subsequent scientific research and clinical data are still needed to support and propose more mechanism of biofilm formation.

## Data availability statement

The data associated with this study has not been deposited into a publicly available repository, and it will be made available on request.

## CRediT authorship contribution statement

**Shebin Zhang:** Conceptualization, Formal analysis, Investigation, Methodology, Project administration, Resources, Writing – original draft. **Yurong Shu:** Writing – original draft. **Weizheng Zhang:** Writing – original draft. **Zhenjie Xu:** Data curation. **Youqiang Li:** Data curation. **Song Li:** Data curation. **Qiwei Li:** Formal analysis. **Rui Xiong:** Data curation, Writing – review & editing. **Yifei Long:** Data curation. **Jianping Liu:** Data curation. **Yunyan Zhang:** Supervision, Formal analysis. **Cha Chen:** Funding acquisition, Project administration, Supervision, Validation, Writing – review & editing. **Yang Lu:** Funding acquisition, Project administration, Resources, Supervision, Validation, Writing – review & editing.

## Declaration of competing interest

The authors declare that they have no known competing financial interests or personal relationships that could have appeared to influence the work reported in this paper.

## Figures and Tables

**figs1:**
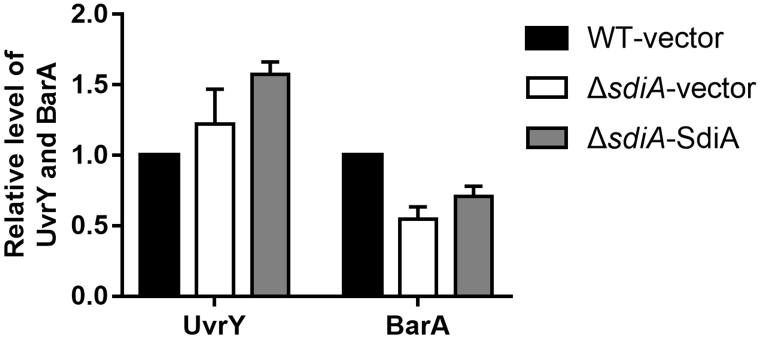


**figs2:**
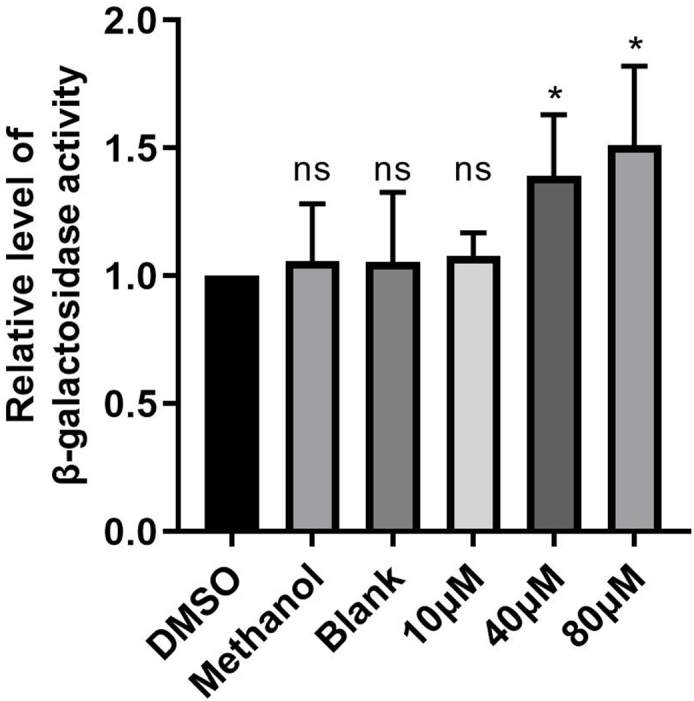


**figs3:**
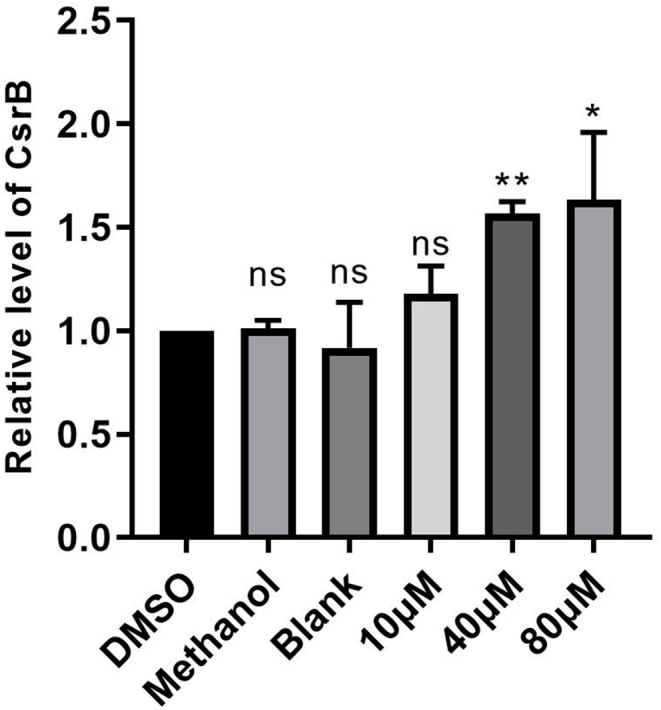


## References

[1] Reisner A., Maierl M., Jorger M., Krause R., Berger D., Haid A., Tesic D., Zechner E.L. (2014). Type 1 fimbriae contribute to catheter-associated urinary tract infections caused by Escherichia coli. J. Bacteriol..

[2] Flemming H.C., Wingender J. (2010). The biofilm matrix. Nat. Rev. Microbiol..

[3] Vakulskas C.A., Leng Y., Abe H., Amaki T., Okayama A., Babitzke P., Suzuki K., Romeo T. (2016). Antagonistic control of the turnover pathway for the global regulatory sRNA CsrB by the CsrA and CsrD proteins. Nucleic Acids Res..

[4] Jackson D.W., Suzuki K., Oakford L., Simecka J.W., Hart M.E., Romeo T. (2002). Biofilm Formation and dispersal under the influence of the global regulator CsrA of *Escherichia coli*. J. Bacteriol..

[5] Dubey A.K. (2005). RNA sequence and secondary structure participate in high-affinity CsrA-RNA interaction. RNA.

[6] Liu M.Y., Romeo T. (1997). The global regulator CsrA of *Escherichia coli* is a specific mRNA-binding protein. J. Bacteriol..

[7] Potts A.H., Leng Y., Babitzke P., Romeo T. (2018). Examination of Csr regulatory circuitry using epistasis analysis with RNA-seq (Epi-seq) confirms that CsrD affects gene expression via CsrA, CsrB and CsrC. Sci. Rep.-UK..

[8] Holmqvist E., Wright P.R., Li L., Bischler T., Barquist L., Reinhardt R., Backofen R., Vogel J. (2016). Global RNA recognition patterns of post‐transcriptional regulators Hfq and CsrA revealed by UV crosslinking in vivo. EMBO J..

[9] Vakulskas C.A., Potts A.H., Babitzke P., Ahmer B.M.M., Romeo T. (2015). Regulation of bacterial virulence by Csr (Rsm) systems. Microbiol. Mol. Biol. R..

[10] Klein G., Raina S. (2017). Small regulatory bacterial RNAs regulating the envelope stress response. Biochem. Soc. T..

[11] Abisado R.G., Benomar S., Klaus J.R., Dandekar A.A., Chandler J.R. (2018). Bacterial quorum sensing and microbial community interactions. mBio.

[12] Dyszel J.L., Soares J.A., Swearingen M.C., Lindsay A., Smith J.N., Ahmer B.M., coli E. (2010). K-12 and EHEC genes regulated by SdiA. PLoS One.

[13] Case R.J., Labbate M., Kjelleberg S. (2008). AHL-driven quorum-sensing circuits: their frequency and function among the Proteobacteria. ISME J..

[14] Styles M.J., Early S.A., Tucholski T., West K., Ge Y., Blackwell H.E. (2020). Chemical control of quorum sensing in E. coli: identification of small molecule modulators of SdiA and mechanistic characterization of a covalent inhibitor. ACS Infect. Dis..

[15] Ma X., Zhang S., Xu Z., Li H., Xiao Q., Qiu F., Zhang W., Long Y., Zheng D., Huang B., Chen C., Lu Y. (2020). SdiA improves the acid tolerance of *E. coli* by regulating GadW and GadY expression. Front. Microbiol..

[16] J.B A., R.R V. (2016). Effect of small chain N acyl homoserine lactone quorum sensing signals on biofilms of food-borne pathogens. J. Food Sci. Technol..

[17] Brackman G., Coenye T. (2015). Quorum sensing inhibitors as anti-biofilm agents. Curr. Pharm. Design..

[18] Lu Y., Li H., Pu J., Xiao Q., Zhao C., Cai Y., Liu Y., Wang L., Li Y., Huang B., Zeng J., Chen C. (2019). Identification of a novel RhlI/R-PrrH-LasI/Phzc/PhzD signalling cascade and its implication in *P. aeruginosa* virulence. Emerg. Microb. Infect..

[19] Baba T., Ara T., Hasegawa M., Takai Y., Okumura Y., Baba M., Datsenko K.A., Tomita M., Wanner B.L., Mori H. (2006). Construction of Escherichia coli K-12 in-frame, single-gene knockout mutants: the Keio collection. Mol. Syst. Biol..

[20] Chen J., Wang Y. (2020). Genetic determinants of Salmonella enterica critical for attachment and biofilm formation. Int. J. Food Microbiol..

[21] Lu Y., Zeng J., Wu B., E S., Wang L., Cai R., Zhang N., Li Y., Huang X., Huang B., Chen C. (2017). Quorum sensing *N*-acyl homoserine lactones-SdiA suppresses *Escherichia coli-Pseudomonas aeruginosa* conjugation through inhibiting *traI* expression. Front. Cell. Infect. Microbiol..

[22] Azam M.W., Zuberi A., Khan A.U. (2020). bolA gene involved in curli amyloids and fimbriae production in E. coli: exploring pathways to inhibit biofilm and amyloid formation. J. Biol. Res. (Thessalon).

[23] Buck L.D., Paladino M.M., Nagashima K., Brezel E.R., Holtzman J.S., Urso S.J., Ryno L.M. (2021). Temperature-dependent influence of FliA overexpression on PHL628 E. coli biofilm growth and composition. Front. Cell. Infect. Microbiol..

[24] Sharma G., Sharma S., Sharma P., Chandola D., Dang S., Gupta S., Gabrani R. (2016). *Escherichia coli* biofilm: development and therapeutic strategies. J. Appl. Microbiol..

[25] Kanamaru K., Kanamaru K., Tatsuno I., Tobe T., Sasakawa C. (2000). SdiA, an Escherichia coli homologue of quorum-sensing regulators, controls the expression of virulence factors in enterohaemorrhagic Escherichia coli O157:H7. Mol. Microbiol..

[26] Yamamoto K., Yata K., Fujita N., Ishihama A. (2001). Novel mode of transcription regulation by SdiA, an Escherichia coli homologue of the quorum-sensing regulator. Mol. Microbiol..

[27] Nguyen Y., Nguyen N.X., Rogers J.L., Liao J., Macmillan J.B., Jiang Y., Sperandio V., Ruby E.G. (2015). Structural and mechanistic roles of novel chemical ligands on the SdiA quorum-sensing transcription regulator. mBio.

[28] Venkatesan N., Perumal G., Doble M. (2015). Bacterial resistance in biofilm-associated bacteria. Future Microbiol..

[29] Garcia-Lara J., Shang L.H., Rothfield L.I. (1996). An extracellular factor regulates expression of *sdiA*, a transcriptional activator of cell division genes in *Escherichia coli*. J. Bacteriol..

[30] Weiss D.S. (2004). Bacterial cell division and the septal ring. Mol. Microbiol..

[31] Shimada K., Ogasawara H., Yamada K., Shimura M., Kori A., Shimada T., Yamanaka Y., Yamamoto K., Ishihama A. (2013). Screening of promoter-specific transcription factors: multiple regulators for the *sdiA* gene involved in cell division control and quorum sensing. Microbiology.

