# Supplementary material for: Quorum sensing N-acyl homoserine lactones-SdiA enhances the biofilm formation of E. coli by regulating sRNA CsrB expression
Source: Heliyon. 2023 Oct 29;9(11):e21658. doi: 10.1016/j.heliyon.2023.e21658 (PMC10651509; doi:10.1016/j.heliyon.2023.e21658)
Supplement: Multimedia component 2 [file mmc2.docx]

**Table S2. Primers used for this study.**

| Primers | Sequence | Purpose |
| --- | --- | --- |
| Promoter-*csrB*-F | CGGGATCCTCCAAATACCCCATCTGGTT | Construction of pQF50-P*csrB* plasmid. |
| Promoter-*csrB*-R | CCCAAGCTTTCGACGAAGATAGAATCGTC | Construction of pQF50-P*csrB* plasmid. |
| *csrB*-F | TATGACCATGATTACGAATTGTCGACAGGGAGTCAGACAA | Construction of CsrB overexpression plasmid. |
| *csrB*-R | ACGACGGCCAGTGCCAAGCTAATAAAAAAAGGGAGCACTG | Construction of CsrB overexpression plasmid. |
| RT_*csrB*-F | TTGCTCCCTGCTCATCCTTG | For qPCR. |
| RT_*csrB*-R | GATTCGGTGGGTCAGGAAGG | For qPCR. |
| RT_*sdiA*-F | TGCAACGGGAAAAGGACAA | For qPCR. |
| RT_*sdiA*-R | GCGGTGTCACTCAGTATTTAATGC | For qPCR. |
| RT_*rpoD*-F | GGGATCAACCAGGTTCAATG | For qPCR. |
| RT_*rpoD*-R | GGTGCCAGATCTTCTTCTGC | For qPCR. |
